# Balancing Host Defense and Viral Tolerance for the Development of Next-Generation Broad-Spectrum Antiviral Agents

**DOI:** 10.3390/pathogens14090911

**Published:** 2025-09-10

**Authors:** Xiujuan Zhao, Ruikun Du, Lijun Rong

**Affiliations:** 1 College of Pharmacy, Heze University, Heze 274000, China; 2 Qingdao Academy of Chinese Medical Sciences, Shandong University of Traditional Chinese Medicine, Qingdao 266122, China; duzi857@163.com; 3 Department of Microbiology and Immunology, University of Illinois at Chicago, Chicago, IL 60612, USA

**Keywords:** host defense, viral tolerance, balance, therapeutic agents

## Abstract

The human immune system has evolved to cope with various virus infections using two major strategies: host defense and viral tolerance. The host defense relies on the innate and adaptive immune responses to control virus replication, while the viral tolerance allows sufficient virus replication in the host with minimal or no clinical signs. Generally, a balanced host defense and viral tolerance can guarantee health from infections, while disturbed immune homeostasis usually results in diseases. It is desirable to develop drugs/therapeutics to enhance the invulnerability of host immune equilibrium to combat viral infections. In this review, we summarize the advanced understanding on mechanisms of both the host defense against and tolerance to virus infections, and therapeutic agents/approaches that work by modulating the balance between host defense and tolerance.

## 1. Introduction

The outcome of virus infection is determined by the intricate interaction between the virus and host [1,2]. The objective of viruses is to replicate themselves, while the hosts attempt to prevent the viral replication/dissemination, and eventually, eliminate these exogenous invasions [3]. To successfully combat the virus infections, it is critical for the host to mount a timely and effective innate immune response to extensively restrict viral replication and dissemination, followed by precise adaptive immunity to clear the invaders effectively [4,5]. On the other hand, the viruses may adopt various strategies to escape or even subvert the host defenses [6,7]. As a consequence, uncontrolled virus replication may trigger excessive immune responses such as cytokine storms, which conversely cause severe tissue damage [8]. Thus, a modulated host immunity is likely preferred, allowing the host to tolerate virus replication to a certain extent with minimal or no clinical signs [9]. Altogether, it is the balanced host defense and viral tolerance that keep the host healthy and free from infection, while a disturbed immune homeostasis leads to diseases.

The human immune system has evolved to cope with various virus infections; nonetheless, individual differences exist due to diverse factors including genetics, age, gender, and varied physiological/pathological status [10]. For instance, the immature neonatal immune system may have a suboptimal defense against infections but high viral tolerance [9]. It has been reported that neonates exhibit a higher proportion of Tregs, which is instrumental in preventing tissue damage by suppressing excessive inflammatory responses [11]. In addition, the activation of the neonatal Toll-like receptor (TLR) signaling pathway is more likely to induce the expression of anti-inflammatory factors (e.g., IL-10) than pro-inflammatory factors (e.g., TNF-α), demonstrating an anti-inflammatory bias [12]. In adulthood, the immune system in general achieves an optimal balance between the rapid recognition of pathogens and the activation of adaptive immunity (e.g., Th1/Th17 cells) via pattern-recognition receptors (PRRs), while limiting excessive inflammation via Tregs and anti-inflammatory factors (e.g., IL-10, TGF-β) [13]. However, immune senescence in elderly individuals may increase the risks of viral infections [14,15,16].

The impacts of gender and physiological/pathological status on the outcome of infection have also been demonstrated. In males, androgens may suppress certain immune responses, resulting in weaker clearance of respiratory viruses [17]. For females, in contrast, estrogens may enhance antiviral immunity (e.g., IFN-α/β production), albeit at a cost of increased risk of autoimmunity [18,19]. In addition, individual immunity may also be disturbed by pathological conditions such as metabolic disorders, leading to increased vulnerability to viral infections [20,21]. Therefore, the outcome of pathogenic infections may vary a lot for different individuals, ranging from asymptomatic to severe diseases, or even death. This necessitates therapeutics as well as other medical care to help people balance defense response and viral tolerance in order to fight or survive infections.

The antiviral drugs can be considered a “foreign aid” of host defense. One strategy for antiviral development is to suppress viral replication, by either blocking the viral factors or cellular components that play essential roles in viral replication, which can be achieved via either direct-acting antivirals (DAAs) or host-targeted antivirals (HTAs) [22]. Indeed, most approved antiviral drugs were developed using this strategy. Nonetheless, due to the high diversity of pathogenic viruses, the effectiveness of these antivirals is usually specific to a single virus or a few closely related viruses. It is desirable, by targeting the conserved mechanism in the replication cycle of multiple viruses, to broaden the antiviral spectrum for these DAAs and HTAs [23].

A different strategy is to develop antivirals that strengthen the host defense against viral infection. These immune modulatory antivirals may possess broad-spectrum antiviral (BSA) properties of effectiveness against a wide range of viruses, as they target commonalities across various viral life cycles rather than specific viral factors. Immune modulatory antivirals have attracted increasing interest in recent years [24]. Of particular note, the therapeutic outcome for these antivirals, especially for antiviral immune modulators, is greatly impacted by the proper timing of administration. The effects of these antiviral immune enhancers may be markedly compromised or even become detrimental if they are used in the late stages of infection or in the case of excessive inflammation [25,26]. Instead, immune suppressors and/or supportive cares are needed to increase the disease tolerance of hosts [27]. Inspired by this, another class of antivirals, or more precisely the “anti-virulence agents”, has been proposed to enhance the host tolerance to viral infections by neutralizing viral and/or host-derived pathogenic signals that cause tissue damage [28].

In this review, we summarize the progress in the development of BSAs by targeting host defense and/or viral tolerance. Moreover, we highlight several feasible strategies for balancing host defense and viral tolerance to develop novel BSAs.

## 2. Immune Modulatory Antivirals Targeting Host Defense

**Figure 1:**
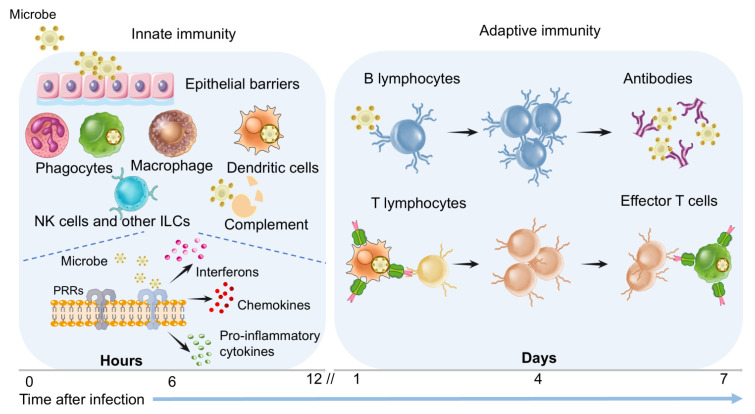
Immune response during viral infections.

The host defense can be broadly classified into two categories: innate immunity and adaptive immunity. Innate immunity is non-specific and serves as the body’s first line of defense against invading pathogens, comprising both constitutive and inducible innate immune responses [29]. The constitutive defenses include the physical barriers (e.g., epithelial barrier), innate immune cells (e.g., macrophages, NK cells), and humoral innate proteins (e.g., complements), while the inducible innate immune responses are mediated by various secretory cytokines, such as interferons, ILs, CSFs, TNFs, and chemokines. In contrast, adaptive immunity targets specific pathogens, requiring proper processing and recognition of individual pathogens by the immune system, which further creates cytotoxic T cells or antibodies specifically designed to protect against invasion (Figure 1) [30,31]. It should be noted that the crosstalk between innate and adaptive immunities has been well documented, and innate immunity plays a pivotal role as the primary activator of adaptive immune responses [32].

We believe it is important to develop immune modulatory agents that enhance either innate or adaptive immunity as antivirals against diverse viruses, especially when these immunities are compromised.

### 2.1. Antivirals Modulating Innate Immune System

#### 2.1.1. Enhancers of the Epithelial Barrier

The epithelial barrier serves as a critical border that prevents invading pathogens from entering tissues. A barrier dysfunction usually results in elevated susceptibility of the host to invading pathogens [33], while enhancement of the barrier can reduce the risk of infection. It is worth noting that surgical masks as medical equipment have been used for a long time to complement the oral and nasal barriers against airborne pathogens [34]. Nasal sprays, mimicking the airway mucus, which constitutes an important part of the airway epithelial barrier, have also been shown to be highly effective against airborne infections [35].

A new strategy for enhancing the epithelial barrier involves targeting the barrier directly. For instance, increasing evidence has demonstrated that flavonoids, as a class of natural bioactive compounds, can markedly enhance the integrity of the airway epithelial barrier [36,37]. Ganapathy et al. reported that alpha-tocopherylquinone, a quinone-structured oxidation product of vitamin E, can reduce intestinal paracellular permeability via an AhR-mediated increase in tight junction barrier-forming protein CLDN3 and an Nrf2-mediated reduction in channel-forming CLDN2 expression [38]. In addition, Sun et al. described a lipid-encapsulated pachypodol that can repair the vascular endothelium–alveolar epithelial barrier in acute lung injury models, thereby exerting a therapeutic effect as well as reducing the risk of new infections [39]. Notably, it has been reported that stem cell exosome nebulization has the ability to reshape the damaged immune barrier of the respiratory tract rapidly, providing a new option for repairing respiratory and lung injuries [40].

Recently, the critical role of intestinal microbiota in maintaining the intestinal mucosal barrier has been reported [41]. First, probiotics such as *Lactobacillus acidophilus*, *Lactobacillus casei*, *Lactobacillus plantarum*, and *Lactobacillus rhamnosus* can lead to a modest enhancement of the intestinal epithelial tight junction barrier [42,43]. Second, carbohydrate metabolism of intestinal flora produces diverse short-chain fatty acids (SCFAs), which can promote proliferation and differentiation of epithelial cells to maintain the mechanical barrier of the intestinal mucosa, and which activate the secretion of mucins by the cup cells to form a dense mucus layer. This helps reduce the adhesion of pathogenic bacteria to the intestinal mucosa. In addition, SCFAs have been shown to display prophylactic and restorative effects on airway epithelial barrier function [44]. Thus, the microbiota is a promising source for the development of epithelial barrier enhancers in the future.

#### 2.1.2. Antivirals Modulating Complement System

The complement system is a central component of innate immunity, clearing pathogens, modulating inflammation, and linking adaptive immunity through a cascade of reactions [45]. First, activation of the complement system leads to the formation of membrane attack complexes (MACs) that directly lyse pathogens. Second, complement fragments, such as C3b, are deposited on the surface of pathogens and act as “phagocytosis signaling” markers, greatly enhancing the phagocytosis efficiency of macrophages and neutrophils. In addition, complement fragments also act as B-cell co-stimulatory signals to enhance antibody production and assist dendritic cells in activating T cells to form specific immune responses. Certain complement components can also function as inflammatory mediators, increasing vascular permeability and promoting local inflammatory responses to limit the spread of pathogens.

Rapid and appropriate activation of the complement system during the early stages of infection contributes to the rapid clearance of pathogens. β-glucan is a complex polysaccharide derived from fungal and yeast cell walls. Preclinical and early clinical trials have shown that in complement system-deficient populations, these polysaccharides act as non-specific immunomodulators, activating not only immune cells but also the complement system, and are currently marketed as antitumor or immunostimulatory agents [46,47]. Recently, Pedersen et al. [48] developed a novel therapeutic modality to direct complement activity to the surface of HIV-1-infected cells. They constructed a bispecific complement engager (BiCE), which can direct complement deposition to the surface of HIV-1-infected cells, leading to complement-mediated killing.

Although the complement system plays a critical role in the host’s fight against invading pathogens, excessive complement activation may conversely lead to tissue damage, uncontrolled inflammation, as well as capillary leakage syndrome [49]. Therefore, accurate diagnosis and precise evaluation are needed before the clinical use of complement system modulators.

#### 2.1.3. Antivirals Modulating Innate Immune Cells

Innate immune cells are a series of immune effector cells formed by organisms during long-term germline evolution. These innate immune cells include monocytes/macrophages, granulocytes (neutrophils, eosinophils, and basophils), dendritic cells (DCs), and natural killer cells (NKs). Additionally, innate immune cells with newly discovered functions, such as innate lymphoid cells (ILCs), γδ T cells, and NKT cells, have been recognized in recent years. The monocytes/macrophages and granulocytes can act against invading pathogens by phagocytosis, while NKs mediate cytotoxic effects against the virus-infected cells. Interestingly, some innate cells, such as macrophages and DCs, can sense the invading pathogens and trigger inducible innate immune responses [50]. These macrophages and DCs also act as antigen-presenting cells, priming a specific adaptive immune response. Importantly, the recruitment and activation of these innate immune cells are finely orchestrated, contributing to immune modulation both during the infection and in subsequent recovery stages [51]. Here, we focus primarily on antiviral strategies that modulate the direct killing mechanisms of innate immune cells.

IL-15 is a pro-inflammatory protein that plays a critical role in activating neutrophils, dendritic cells, and macrophages, and which is essential for the development and survival of NK cells and CD8+ T cells [52]. ALT-803, a novel IL-15 super-agonist, has been shown to boost NK and CD8+ T-cell responses to HIV infection, promoting the rapid expansion of both cell populations in vitro and in vivo [53]. These IL-15 agonists are promising immunomodulating agents to elicit effective control of HIV/SIV replication [54].

Polysaccharides represent another class of potent immunostimulants. It has been reported that edible fungal polysaccharides can regulate macrophage polarization, dendritic cell maturation, and T/B-cell activation, achieving synergistic enhancement of both innate and adaptive immunity [55,56]. *Ganoderma lucidum* polysaccharides have demonstrated the ability to promote the maturation and function of dendritic cells and the initiation of a DC-induced immune response. In addition, oral administration of *Ganoderma lucidum* extracts can significantly increase the phagocytic capacity of macrophages and the activity of NK cells [57]. Chitosan, an animal-derived polysaccharide, augments the antiviral immune responses by stimulating immune cells, increasing the number of phagocytes, and promoting the migration of neutrophils [58]. Thus, polysaccharides have attracted increasing attention due to their high activity, low toxicity, and availability for future antiviral development.

Resveratrol is a class of phenolic compounds, mainly found in the pulp, peel, seeds, and stems of red grapes. It exhibits antiviral effects against various viruses by enhancing the cytotoxic activity of NK cells, increasing the phagocytic rate of macrophages, and facilitating pathogen clearance [59]. In addition, it helps reduce cytokine storms and limits parenchymal damage in the lungs. These properties have been documented in infections with influenza virus, respiratory syncytial virus, human rhinovirus, and SARS-CoV-2 [60].

Sialic acid-binding immunoglobulin-like lectins (Siglecs) are a family of proteins expressed on most immune cells [61]. Siglec-blocking antibodies can enhance immune function against virus-infected cells [62]. For example, anti-Siglec-9 antibody enhances NK cytotoxicity against HIV-infected targets and reverses abnormal NK-cell function during HBV infection [63].

In addition to the NK cells and macrophages mentioned above, neutrophils also play a significant role in the fight against viral infections. Neutrophil dysfunction may lead to more severe infection symptoms [64]. Therefore, neutrophil-mediated innate immunity is also an important area for antiviral research.

#### 2.1.4. Antivirals Modulating Inducible Innate Antiviral Immunity 

When pathogens invade, their conserved structures, known as the pathogen-associated molecular patterns (PAMPs), can be recognized by pattern-recognition receptors (PRRs) on the surface or inside innate immune cells [65], triggering cascades of signaling pathways and the production of stimulatory cytokines, including interferons (IFNs), chemokines, and various pro-inflammatory cytokines [66]. Among these cytokines, IFNs directly promote the establishment of an antiviral status through the JAK/STAT signaling pathway in both infected and neighboring cells through autocrine and paracrine mechanisms, respectively. Indeed, the IFN response has become one of the most attractive and promising targets for the development of novel BSAs.

##### Recombinant IFN and IFN Analogues

One strategy to complement the host IFN response is the use of recombinant IFNs, which have been approved for treating both acute and chronic infections caused by diverse viruses, including RSV, HBV, HCV, and HPV [67]. However, with the widespread application of recombinant IFNs, mild-to-moderate adverse drug reactions (ADRs) have been observed, such as eruptions, fever, muscle soreness, anaphylaxis, and abnormal liver function. In addition, a strict treatment window (only during the early stages of infection) and an appropriate dosage are critical for the effective use of recombinant IFNs. This is particularly important when treating respiratory viral infections, since excessive IFN may trigger an inflammatory storm [68].

Recombinant interferon α-2b (rhINF-α-2b) is an antiviral treatment used clinically. However, like most therapeutic proteins, it has several shortcomings, such as a relatively short half-life, a narrow therapeutic index, high circulating drug fluctuations, and rapid degradation. To prolong the half-life of interferon while maintaining its biological activity, a long-acting pegylated interferon α-2a has been developed and approved by the U.S. FDA for the treatment of HBV patients [69].

Small-molecule peptide cytokine mimetics have advantages as antivirals due to their relatively small molecular weight, ease of synthesis, non-antigenicity, and low toxicity. For example, two IFN α-2b mimetic peptides with antiviral activity were derived from a phage-display heptapeptide library using a novel functional selection method [70]. This approach provides a promising strategy for the development of small-peptide antivirals.

##### Agonists of IFN Production

Another promising strategy for developing IFN-based antivirals focuses on boosting the production of intrinsic IFNs. It is reasonable to envision that pattern-recognition receptors (PRRs) (TLRs, RLRs, etc.), adaptors (MyD88, TRIF, MAVS), and critical downstream transcription factors (IRFs, etc.) could all serve as targets when developing agonists for IFN production.

PRRs play a crucial role in detecting viral nucleic acids and initiating innate immune defenses, including the IFN production pathway. Direct activation of PRRs by specific agonists triggers downstream signaling cascades, leading to potent antiviral responses. Furthermore, enhancing PRR expression can also amplify this protective pathway [71]. For example, TLR7 and TLR8, located in endosomes, recognize viral single-stranded RNA. Synthetic agonists like the imidazoquinoline resiquimod (R848) directly activate TLR7/TLR8, demonstrating significant potential as antiviral adjuvants [72]. STING (Stimulator of Interferon Genes) is another essential PRR that senses cytosolic cyclic dinucleotides; meanwhile, agonists like diABZI potently activate STING, inducing IFN production and conferring broad-spectrum antiviral activity against coronaviruses like SARS-CoV-2 [73].

Adaptor proteins transduce signals from activated PRRs to downstream kinases and transcription factors. Promoting the activity or abundance of these adaptors can effectively enhance IFN production. For instance, upregulation of MyD88 during many virus infections is associated with a decrease in antiviral type I IFN induction, and research indicates that the MyD88 inhibitor compound 4210 exhibits broad-spectrum antiviral activity by upregulating type I interferon. This activity involves promoting TRIF’s interaction with downstream effectors to amplify IFN responses [74].

Interferon regulatory factors (IRFs), particularly IRF3 and IRF7, are master transcription factors that directly regulate type I and III IFN gene expression upon activation. Compounds that promote IRF phosphorylation and activation offer a strategic approach to bolster antiviral immunity. Gilteritinib (already approved for leukemia treatment) has been shown to facilitate IRF7 phosphorylation, exhibiting potent broad-spectrum antiviral activity [75]. Other compounds, such as the isoflavones KIN 100 and KIN 101, act as specific agonists of innate immune signaling pathways and cause activation of the IRF-3 transcription factor [76].

##### Enhancers of IFN Function

After secretion, IFNs are recognized by interferon receptors (IFNARs) on the surfaces of both infected and surrounding uninfected cells, initiating the Janus kinases-signal transducer and activator of transcription (JAK/STAT) pathway. This leads to the expression of various interferon stimulated genes (ISGs), most of which possess antiviral properties. The IFN signaling pathway has thus become an attractive target for BSA development, and numerous activators or enhancers of JAK/STAT signaling have been reported [77,78].

Emodin (1,3,8-trihydroxy-6-methyl anthraquinone) was shown to activate JAK/STAT signaling and improved the antiproliferative effect of IFN-α [79]. 5-Hydroxymethylfurfural (5-HMF), a heterocyclic furan-containing aldehyde generated by the Maillard reaction, significantly upregulates the phosphorylation levels of STAT1 and STAT2 in macrophages stimulated by IFN-α/β during VSV infection [80]. Recently, we identified kaempferide as a novel enhancer of the JAK/STAT pathway, by prolonging the duration of IFN signaling. Notably, kaempferide can synergize with both intrinsic and exogenous IFNs, exerting significant BSA activity [81].

### 2.2. Antivirals Modulating Adaptive Immune System

Unlike innate immunity, which exerts common antiviral properties immediately or soon after viral infection, adaptive immune responses require specific recognition and processing of viral-derived antigens, conferring delayed but stronger protection against specific pathogens on the host [31]. In addition, adaptive immunity has been well characterized to possess immunological memory, which provides a long-lasting protection of the host from the previously encountered pathogens [82]. Indeed, vaccines have been developed successfully to combat or even eliminate certain viruses [83]. Essentially, vaccines simulate the invasion of a pathogen (e.g., virus or bacterium), training the immune system to recognize and remember the characteristics of the pathogen without triggering a real disease. This allows for rapid activation of defense mechanisms upon real infection, thereby preventing illness or alleviating symptoms. Vaccines have significantly reduced the incidence and mortality rates of infectious diseases, highlighting their profound impact on public health. Nonetheless, vaccines have their limitations; for example, (1) vaccination is generally effective only when administered at least a couple of months prior to the infection, and (2) there are limited or no options in terms of vaccines for many infectious viruses. Therefore, it is important to promote or augment the adaptive immune responses to the infections without pre-existing immunity.

#### 2.2.1. Modulators of T-Cell Immunity

When pathogens invade the host, they are processed through antigen presentation to form antigen-MHC molecular complexes, which are presented to T lymphocytes for recognition [84]. Upon antigen stimulation, T cells undergo activation, proliferation, and differentiation to become effector T cells (mainly CTL and CD4Th cells), which execute immune functions. Among them, CTL cells directly induce apoptosis of infected cells and inhibit viral replication by releasing perforin and granzyme; meanwhile, CD4Th cells secrete cytokines such as IL-2 and IFN-γ, which promote CTL proliferation, enhance macrophage phagocytosis, and assist B cells in producing neutralizing antibodies [85]. Antiviral drugs that enhance T-cell immunity can significantly improve the host’s ability to clear viruses by activating the immune response in time and strengthening the intensity of immune killing.

Scutellaria baicalensis glycosides exhibit anti-inflammatory and immunomodulatory properties. Astragaloside II enhances T-cell activation by modulating the activity of CD45 PTPase, thereby exerting antiviral effects [86,87]. Acteoside, a natural phenylpropanoid glycoside derived from Kuding Tea, enhances IFN-γ production in mouse lymphocytes in a dose-dependent manner, particularly in the CD4+ and CD8+ subsets of T lymphocytes [3,88], contributing to its antiviral activity. Alisporivir stimulates antigen presentation by upregulating MHC-I surface expression, thereby promoting antigen-specific CD8+ T-cell activation. This immunostimulatory function may further contribute to the antiviral activity [89]. Azvudine, also known as FNC, is a thymus-homing anti-SARS-CoV-2 drug used in treating COVID-19 patients [90]. Azvudine and its metabolites are enriched in the rat thymus and further activate key immune cells, mainly CD4+ and CD8+ T cells. FNC treatment enhances the defense response against SARS-CoV-2 infection and promotes the immune response of T cells in the thymus of rhesus monkeys [91].

Viral infection can increase the expression of PD-1 and PD-L1 on the surface of antigen-specific T cells [92], inhibiting the proliferation of CD4+ and CD8+ T cells and reducing the secretion or diffusion of cytokines such as IL-2 and IFN-γ. They can also cause a reduction (or even failure) in the immune function of specific T lymphocytes. This weakens the host’s anti-infection immune response, leading to damage to the target organ and ultimately causing persistent viral infection and related diseases [93,94]. These findings suggest that the PD-1/PD-L1 pathway may be one of the main causes of chronic viral infections and the chronic nature of the disease. The PD-1/PD-L1 pathway has been associated with chronic viral infections involving T cells in HIV [95], HBV [96], HCV [97], and Hantavirus [98] infections. In chronic viral infections, blocking PD-1 with antibodies allows CD8+ T cells to be reactivated, proliferate, and differentiate, restoring their ability to kill viruses and reducing viral titers [99].

#### 2.2.2. Modulators of B-Cell Immunity 

B cells recognize viral antigens through surface receptors (BCR) and can be activated and differentiated into plasma cells and memory B cells with the assistance of T cells [100]. Plasma cells secrete specific antibodies (such as IgG/IgM), which directly eliminate pathogens by neutralizing viruses, regulating phagocytosis, activating complements, and mediating antibody-dependent, cell-mediated cytotoxicity. Meanwhile, B cells, as antigen-presenting cells, present viral antigens to T cells via MHC-II, thereby amplifying the immune synergy effect. Memory B cells survive in lymphoid tissue for a long time and can rapidly initiate an efficient secondary response when encountering the same virus. Therefore, developing therapeutic strategies that target and enhance B-cell activity can promote antibody production and improve the antiviral immune response [101].

Compound MGN1703 (lefitolimod) has been shown to modulate human lymph node B cells in vivo. MGN1703 administration increased B-cell differentiation and secondary B-cell follicle formation, and it induced elevated plasma IgG secretion in individuals infected with HIV-1 [102]. Bushen Formula, an effective traditional Chinese medicine for chronic hepatitis B, stimulates B-cell growth and modulates B-cell subsets, which play a crucial role in the clearance of HBV [103]. In addition, many polysaccharides derived from traditional Chinese medicines have been shown to possess immunomodulatory effects [104]. For example, ginsenosides, the major active components of ginseng, are known to modulate blood pressure, metabolism, and immune function, and they have been used to treat different diseases. Park et al. [105] suggested that ginsenosides Rg1 and 20(S)-Rg3 can promote the differentiation of B cells into IgA-producing cells. Notably, Rg3 exhibits the broadest spectrum of antiviral efficacy against HVJ, RV, HSV, HBV, HCV, and GCRV infections [106]. Astragalus polysaccharides (APSs) can stimulate the proliferation of B cells and macrophages and increase cytokine production [107], which is also one of the key mechanisms through which APS exerts its antiviral effects [108]. The emergence of these herbal medications has opened up new avenues for the control and treatment of viral infections.

Interestingly, recent studies have identified acetylcholine (ACh)-secreting B cells as key regulators in the early regulatory cascade controlling lung tissue damage after viral infection. These cells reduce inflammatory damage at the expense of transiently enhanced viral replication during early infection stages, offering new perspectives for antiviral therapeutic strategies involving α7-nAChR agonists [109].

## 3. Immune Modulatory Antivirals Targeting Viral Tolerance

Most viruses we encounter are quickly eliminated by the body’s immune system and cause few symptoms, but a small number of them are pathogenic and lead to mild to severe diseases or even death. The outcome of these pathogenic viral infections is determined not only by the efficiency of host defense but also by the host’s ability to tolerate the existence of the virus. For instance, some pathogenic viruses can escape host immunity, allowing them to replicate uncontrollably. Such high viral loads might evoke excessive immune responses, often characterized by disproportionate synthesis and release of large amounts of cytokines, including tumor necrosis factor-α (TNF-α), interleukin 1 (IL-1), interleukin 6 (IL-6), and interferon-γ (IFN-γ). This hypercytokinemic (also known as cytokine storm) condition is intimately linked with the progression of viral diseases and associated complications and mortality [110,111]. Under such conditions, direct antiviral drugs or targeted enhancement of the host’s antiviral immunity are usually ineffective, and they may even exacerbate the condition. Instead, suppressing the exaggerated immune response and surviving the life-threating condition are the best options [112].

Viral tolerance is a survival strategy in which the host, when confronted with viral infection, regulates its own physiological and immune responses to reduce tissue damage caused by the virus rather than completely eliminating the virus [113]. Clinically, several classes of approved immune suppressors are available to enhance the tolerance of viral infections [114]. However, our arsenals to treat severe viral diseases are very limited. The mortality rate among critically ill patients with SARS-CoV-2 pneumonia is considerable. Multicenter retrospective studies indicate that the mortality rate of severe cases is ~ 60% [115,116]. In this section, we describe the existing options and elaborate on the state-of-the-art advancements in the development of antiviral (or more precisely “anti-virulence”) agents that enhance host tolerance to viral infections.

### 3.1. Glucocorticoids (e.g., Dexamethasone and Hydrocortisone)

Glucocorticoids, a class of steroid hormones secreted by the zona fasciculata of the adrenal cortex, are the most widely used anti-inflammatory drugs. They are non-specific anti-inflammatories with powerful anti-metamorphic and anti-shock characteristics [117,118]. In long-term clinical treatment, glucocorticoids are primarily used to control excessive inflammatory responses in autoimmune diseases (e.g., rheumatoid arthritis, systemic lupus erythematosus) and chronic inflammatory conditions (e.g., asthma, inflammatory bowel disease). They can significantly alleviate tissue erythema, pain, and organ dysfunction through sustained suppression of inflammatory cascades [119].

For excessive inflammatory responses caused by viral infections (such as cytokine storms), glucocorticoids significantly reduce the release of pro-inflammatory factors including IL-6 and TNF-α by inhibiting inflammatory pathways like NF-κB. They also attenuate immune cell activity, thereby rapidly controlling tissue damage and edema [120]. Particularly in severe diseases like COVID-19, they have been shown to significantly reduce mortality [121]. However, their side effects need to be vigilantly watched: short-term use may delay viral clearance and increase the risk of secondary bacterial or fungal infections; long-term application can lead to metabolic disorders (such as high blood sugar, high blood pressure, central obesity), osteoporosis, and even femoral head necrosis, and they may also induce peptic ulcers or psychiatric symptoms [122,123]. Clinically, the indications must be strictly controlled and limited to severe patients only.

### 3.2. Monoclonal Antibodies Specific to Pro-Inflammatory Cytokines

Pro-inflammatory factors play a key role in regulating and coordinating immune responses during the early stages of viral infection, helping the body effectively defend against viral attacks. However, excessive or inappropriate pro-inflammatory cytokine release may lead to overactivation of the immune system, triggering inflammatory diseases or immune-mediated injury. Therefore, the regulation of appropriate pro-inflammatory cytokine release is essential for controlling viral infections and maintaining immune homeostasis [124].

IL-6 and IL-1β are key pro-inflammatory cytokines and major drivers of excessive inflammation and tissue damage. Anti-IL antibodies do not directly kill viruses or enhance antiviral immunity; instead, they target the uncontrolled host immune response. By blocking the binding of IL to its receptor, they inhibit the activation of downstream signaling pathways such as JAK-STAT3. There are two classes of approved IL-6 inhibitors: anti-IL-6 receptor monoclonal antibodies (e.g., tocilizumab) and anti-IL-6 monoclonal antibodies (e.g., siltuximab) [125]. Additionally, antibodies such as sarilumab directly target the IL-6 receptor and block IL-6 signaling. Canakinumab is an IL-1β neutralizing antibody that reduces hyperinflammation by binding and antagonizing inflammatory mediators, such as IL-1β and IL-1α, and IL-1 decoy receptors [126]. It has proven to be helpful in COVID-19 patients suffering from hyperinflammatory syndrome [127]. It is worth noting that such treatments are typically reserved for severe cases with clear evidence of excessive inflammation [128].

### 3.3. Natural Product-Derived Antiviral Immune Suppressors

Owing to their immense structural diversity and remarkable biological activities, natural products represent a rich source for discovering novel immunosuppressants with high potency and novel mechanisms of action. In recent years, increasing attention has been directed toward exploring effective immunomodulatory and anti-inflammatory agents from the herbal compendium of traditional medicines. Of the various bioactivities of natural plant products that have been reported to date, anti-inflammatory properties are among the most frequently documented, primarily identified in terpenoids [129], polyphenols [130], alkaloids [131], and flavonoids [132].

Berberine, an isoquinoline alkaloid, directly inhibits the function and differentiation of pro-inflammatory Th1 and Th17 cells, and it indirectly reduces Th cell-mediated inflammation by modulating or inhibiting cells involved in autoreactive inflammation [133]. A variety of viruses activate or inhibit NLRP3 inflammatory vesicles via viral particles, proteins, and nucleic acids. It has been demonstrated that berberine can suppress influenza virus-induced activation of the NLRP3 inflammasome in macrophages [134,135]. This effect is achieved by inducing mitochondrial autophagy and reducing mitochondrial ROS, alleviating the excessive inflammatory responses during viral infections.

Luteolin, a natural flavonoid compound derived from vegetables, fruits, and herbs, exhibits potent anti-inflammatory activity. It can alter the M1/M2 polarization of macrophages, thereby playing an anti-inflammatory role via downregulation of p-STAT3 and upregulation of p-STAT6 [136,137].

### 3.4. Cellular Therapy-Based Antiviral Strategy for Viral Tolerance Enhancement

The strategy of enhancing virus tolerance based on cellular therapy represents an emerging therapeutic approach. This method involves collecting a patient’s own immune cells, expanding them in vitro to increase their numbers by several orders of magnitude, and enhancing their antigen-specific cytotoxicity. The activated cells are then reinfused into the body to eliminate malignant cells, overcome immune tolerance, and enhance the body’s immune capacity.

Mesenchymal stem cell (MSC) therapy is a form of cellular treatment based on modulating inflammatory responses and promoting tissue repair and regeneration [138]. It can effectively mitigate severe inflammatory responses caused by viruses, reduce lung injury, and improve lung function. MSC therapy has been applied in the treatment of viral infections, as it can reverse cytokine storms and inhibit the overactivation of the immune response [139]. Its primary mechanisms include suppressing pro-inflammatory factor production by NK cells, inhibiting dendritic cell (DC) differentiation, maturation, and antigen-presenting capacity, and modulating macrophage polarization. In patients with COVID-19, the cytokine storm occurs after overproduction of inflammatory agents by the immune system. MSCs help regulate the release and activity of cytokines under these conditions through endogenous repair and the compensatory properties of stem cell-derived products. A double-blind controlled phase II trial recruited severe COVID-19 patients with lung damage to be treated with infusion of MSCs. The ratio of solid component lesion volume remarkably decreased in the patients treated with umbilical cord MSCs without obvious side effects, suggesting that MSC exosomes could offer a potential option for treating COVID-19 [140]. Furthermore, MSC infusion has been demonstrated to protect alveolar epithelial cells and promote neovascularization [141].

Since the outbreak of COVID-19, scientists have considered using NK cells as an effective cell therapy for treating patients with COVID-19 [142]. The chimeric antigen receptor (CAR) is a genetically engineered receptor widely used in the treatment of various cancers. Engineered NK cells expressing CAR molecules can specifically target cells expressing viral antigens. As a result, CAR-NK-engineered cells have been proposed as a novel therapeutic approach for COVID-19 [143].

## 4. Novel Strategies of Antiviral BSA Development by Balancing Host Defense and Virus Tolerance

Antiviral immune modulation by enhancing host defense or viral tolerance represents two complementary strategies. However, as previously mentioned, host immunity is double-edged sword and both strategies have limitations. On the one hand, when immunostimulants are used for antiviral therapy in clinical practice, particular attention must be paid to the timing of administration [144]. For instance, IFN-I must be administered promptly after infection to be effective, while administering IFN therapy late in the course of infection may result in an inability to suppress viral replication and an increased incidence of adverse effects. On the other hand, immune tolerance suppresses inflammation to a degree that may slow the rate of viral clearance, and the efficacy of such antiviral effects can vary widely among individuals.

An ideal immune modulator, such as BSA, should possess bidirectional regulatory effects: at early stages of infection or in the case of immune-defective patients, the immune modulators can directly enhance the host’s immune defenses by activating specific antiviral immune pathways or enhancing the clearance ability of immune cells; conversely, in later stages of viral infection or in the condition of hypercytokinemia, the immune modulators can significantly increase the host’s tolerance to the virus, by modulating excessive inflammatory responses and reducing immunopathological damage. This combination of ‘offensive and defensive’ properties should allow these immune modulators to protect the host more effectively from diverse viral infections (Table 1).

Traditional herbal medicines, characterized by multi-components, multiple targets, and integrated mechanisms of action, may have potential as BSAs by performing immune modulation in a bidirectional manner.

*Scutellaria barbata* D. Don (SBD) is a widely used herb in traditional Chinese medicine (TCM) with diverse pharmacological activities, including anticancer, antioxidant, anti-inflammatory, antiviral, and antibacterial effects. Extracts of *Scutellaria barbata* D. Don have been shown to alleviate SARS-CoV-2-induced acute lung injury by inhibiting viral replication and bidirectionally modulating immune responses. SBD balances both the benefits and risks of immune modulations carefully through a mode of bidirectional regulation [145].

Licorice is one of the most widely used medicinal plants globally for treating various diseases. Its key component, glycyrrhizic acid, acts as an effective antioxidant and exhibits anti-inflammatory, antiviral, antitumor, and immunomodulatory properties [146,147]. Glycyrrhetinic acid (GA) has been found to inhibit HIV and induce interferon production. It also enhances the NK-cell activity and increases the number of CD4+ T lymphocytes [148]. Furthermore, GA may enhance the immune system by inducing CD4+ anti-suppressor T cells, thereby increasing resistance to herpesvirus infections in immunocompromised individuals [149]. Licorice has been shown to significantly upregulate the expression of the IFN-γ gene in cells infected with H1N1 and downregulate the expression of the inflammatory cytokine TNF-α gene, thereby alleviating the inflammatory response induced by flu H1N1 and reducing the immune damage of the host cells.

Platycodon grandiflorus (PG) is a perennial herb known for its effectiveness in treating respiratory tract infections. As an antiviral agent, PG can reduce inflammation, enhance T-cell and macrophage function, and bolster host immunity. PG binds to TRAF6, reducing its K63-linked ubiquitination and inhibiting the activation of the MAPK and TAK1/IKK/NF-κB pathways [150]. This downregulates the overactivated inflammatory responses and immune cell infiltration, improving survival in influenza infection. Additionally, PG enhances the immunogenicity of viruses and the activity of cytotoxic T lymphocytes, phagocytes, and natural killer cells through cellular and humoral immune responses [151,152].

These natural medicines exhibit multitarget properties that align closely with the theory of bidirectional immune regulation. They offer a promising direction for developing safe, broad-spectrum agents for treating viral infections and related inflammatory diseases. Based on this, we can further analyze the pharmacological mechanisms and material basis of these bidirectional immunomodulatory traditional Chinese medicines, and we can develop modern antiviral drugs that fulfill the ’balancing host defense and tolerance’ strategy.

## 5. Conclusions and Future Perspectives

This review summarizes the dynamic balance between host defense and viral tolerance—two key strategies of the immune system for coping with viral infections—and their potential applications in antiviral therapy. Current drug development has primarily focused on either enhancing host defense or promoting viral tolerance. However, there are limitations to this approach: the immune stimulation may exacerbate the inflammatory responses, while excessive suppression of immunity or inflammation can delay viral clearance.

Based on the ’balancing host defense and tolerance’ strategy proposed here, future antiviral drug development may, on the one hand, involve the development of bidirectional immunomodulators and the exploration of the multitargeting properties of natural products (e.g., glycyrrhizic acid and platycodonis saponin). On the other hand, we should focus on precisely regulating a bidirectional immune balance and developing drug combinations that mimic the ’high antiviral–low inflammation’ characteristics of the host’s unique immune balance mechanism. This approach will offer a more balanced and safer therapeutic strategy for treating emerging viral infections.

## Figures and Tables

**Table 1:** Several typical therapeutic agents based on different antiviral strategies.

Antiviral Strategy	Target	Therapeutic Agent	Mechanism of Action	Antiviral Activity	Ref.
Host defense	Epithelial barrier	Flavonoids	Enhance integrity of airway epithelial barrier	AIV, IAV	[36,37]
		Probiotics	Enhance intestinal epithelial tight junction barrier, produce SCFAs	IAV, SARS-CoV-2, HBV, HSV, RSV,	[41,42,43,44]
	Complement System	β-glucan	Activate the complement system	SARS-CoV-2	[46,47]
	Innate Immune Cells	ALT-803	IL-15 super agonist, boost NK and CD8+ T-cell responses to infection	HIV, SIV	[53,54]
		Resveratrol	Enhance NK cytotoxicity and macrophage phagocytosis	IAV, RSV, SARS-CoV-2	[59,60]
	Inducible Innate Immunity	Recombinant IFNs	Establish antiviral status	RSV, HBV, HCV, HPV	[67,68,69]
		R848	Activate TLR7/TLR8	HIV	[71]
		Compound 4210	Activate IRF3/IRF7 and promote IFN-I responses	EBOV, MbV, CV	[74]
		Gilteritinib	Facilitate IRF7 phosphorylation	SARS-CoV-2, IAV, WNV, HIV	[75]
		Kaempferide	Enhance JAK/STAT pathway	SFTSV, CCHFV	[81]
Host defense	T-cell Immunity	Astragaloside II	Enhance T-cell activation	DENV	[86,87]
		Alisporivir	Enhance MHC-I surface expression Promote antigen-specific CD8+ T-cell activation	HCV	[89]
		Azvudine	Activate T-cell phosphorylation	HIV, SARS-CoV-2	[90,91]
	B-cell Immunity	Compound MGN1703	Enhance B-cell differentiation and function	HIV	[102]
		Bushen formula	Regulate B-cell subsets	HBV	[103]
		Astragalus polysaccharides	Activate B lymphocytes and macrophages	SARS-CoV-2	[107,108]
Viral tolerance	Inflammatory Responses	Glucocorticoids	reduce pro-inflammatory factor release, inhibit inflammatory pathways	Non-specific	[117,118,119,120,121]
		Monoclonal antibodies (tocilizumab, siluximab)	Block IL-6 binding to receptor, inhibit JAK-STAT3 signaling	SARS-CoV-2	[125]
		Berberine	Inhibit the activation of the NLRP3 inflammasome	IAV	[134,135]
		Mesenchymal stem cell therapy	Suppress pro-inflammatory factor production	SARS-CoV-2	[139,141]
Balance host defense and virus tolerance	------	*Scutellaria barbata* D. Don	Attenuate viral replication and alleviate inflammatory responses	SARS-CoV-2	[145]
		Glycyrrhetinic acid	Enhance immune system, alleviate inflammatory response	SARS-CoV-2, HIV, IAV, HSV	[146,147,148,149]
		Platycodon grandiflorus	Reduce inflammation, enhance T-cell and macrophage function, bolster host immunity	IAV, HBV	[150,151,152]

Abbreviations: AIV, avian influenza virus; CCHFV, Crimean-Congo hemorrhagic fever virus; CV, coxsackievirus; DENV, dengue virus; EBOV, Ebola virus; HBV, hepatitis B; HCV, hepatitis C virus; HIV, human immunodeficiency virus; HSV, herpes simplex virus; IAV, influenza A virus; MbV, monkey B virus; RSV, respiratory syncytial virus; SFTSV, severe fever with thrombocytopenia syndrome bunyavirus; WNV, West Nile virus.

## Data Availability

Data sharing not applicable. No new data were created or analyzed in this study.
